# ChatGPT for diabetes education: potential, accuracy, and accessibility in patient support

**DOI:** 10.3389/fdgth.2026.1768843

**Published:** 2026-04-13

**Authors:** Mohammad Abuzar, Sandeep Rai, Tabreskhan Pathan, Foorkan Fakki, Shariq Syed

**Affiliations:** 1 Anjuman-I-Islam’s Kalsekar Technical Campus School of Pharmacy, New Panvel, India; 2 Department of Internal Medicine, Incharge- Diabetes Speciality Centre, MGM Institute of Health Sciences, Navi Mumbai, India; 3 Diabetologist-Apollo Hospital, CBD Belapur, Navi Mumbai, India

**Keywords:** AI in healthcare, artificial intelligence, blood glucose control, ChatGPT, diabetes mellitus, patient education

## Abstract

**Background:**

Diabetes mellitus is a chronic metabolic disease with rising global prevalence. Adequate patient education is essential to encourage self-management and reduce complications. Artificial intelligence applications such as ChatGPT have emerged as potential supplementary resources for patient education alongside the broader integration of technology in healthcare.

**Methods:**

A cross-sectional evaluation was conducted using ten frequently asked questions (FAQs) on diabetes, selected from the Diabetic Association of India and the International Diabetes Federation. ChatGPT-4o (accessed via the web interface in March 2025) generated responses to each question in separate, stand-alone chat sessions to simulate typical patient interactions. Five board-certified endocrinologists (diabetologists) with a mean clinical experience of ≥10 years independently evaluated the responses using a 4-point Likert scale across five domains: overall quality, content accuracy, clarity, relevance, and trustworthiness. Final domain scores were computed as the mean of all five raters’ scores. Readability was assessed using the Flesch Reading Ease Score (FRES) and Flesch-Kincaid Grade Level (FKGL). All readability analyses apply exclusively to the English-language outputs generated in this study.

**Results:**

The mean FRES was 38.19 and the mean FKGL was 16.87, indicating a reading level appropriate for college-educated individuals and substantially above the recommended sixth-grade benchmark for patient health materials. Mean response length was 300 ± 100 words across the ten prompts. Expert ratings were generally high: aggregated mean scores (±SD) were 4.0 (±0.0) for content accuracy and overall quality, 3.98 (±0.10) for relevance, and 3.9 (±0.20) for clarity and trustworthiness. No clinically inaccurate statements were identified by the raters; however, the high scores and narrow score range indicate a potential ceiling effect that limits discrimination between responses. Raters expressed concern about linguistic complexity, which may impede comprehension among patients with limited health literacy.

**Conclusions:**

ChatGPT-4o generated generally accurate and relevant diabetes education content, suggesting potential as a supplementary tool in diabetes care. However, the high reading-level complexity, small evaluation scope (ten prompts, one model, one session), and English-only assessment limit the generalisability of these findings. AI-generated content should supplement, not replace, clinician-led education. Future work should address language simplification, multilingual evaluation, and longitudinal assessment of patient outcomes.

## 1 Introduction

Generative artificial intelligence (AI) tools, including the Chat Generative Pre-trained Transformer (ChatGPT; OpenAI, USA), have attracted considerable attention owing to their sophisticated natural language processing capabilities and potential applications across healthcare and education. These tools belong to the class of large language models (LLMs), trained to recognise contextual relationships between words and sentences and to generate contextually appropriate responses to user queries (1, 2). Rapid advances in neural network architectures have expanded the applicability of these models across diverse domains (3). Current versions of ChatGPT are freely or inexpensively accessible to the general public and are trained on large text corpora that include medical and educational content (4).

Several LLMs are currently available, including Google's PaLM, Anthropic's Claude, and Meta's LLaMA. Each model differs in architecture, training data, and response characteristics. ChatGPT was selected for the present study because of its widespread public accessibility, its demonstrated performance across healthcare communication tasks in prior literature, and its broad training corpus encompassing medical and educational material (5, 6). This selection does not imply superiority over other models; comparative evaluations across multiple LLMs represent an important direction for future research.

Diabetes mellitus represents a major and growing public health burden. Approximately 38.4 million Americans (11.6% of the population) are affected (7). In India, the ICMR-INDIAB study estimated that over 101 million adults aged 18 years or older have diabetes, with an additional 135 million in a pre-diabetic state (8). The two principal forms are type 1 diabetes (T1D), an autoimmune condition characterised by destruction of pancreatic beta cells, and type 2 diabetes (T2D), primarily associated with insulin resistance and influenced by lifestyle, environmental, and genetic factors. Gestational diabetes and other less common forms also contribute to the overall disease burden (9).

Accessible and reliable patient education is central to diabetes management, enabling individuals to recognise symptoms, understand disease mechanisms, and adopt strategies to prevent complications (10). The integration of AI into healthcare has created new opportunities for scalable, personalised patient education and self-management support (11). ChatGPT may serve as a supplementary resource for delivering real-time health information; however, rigorous evaluation of the accuracy, relevance, and readability of AI-generated educational content is essential before such tools can be responsibly recommended for patient use (12).

Patients and caregivers increasingly rely on online resources for health information outside clinical settings. The quality and accuracy of such information, including AI-generated content—varies considerably. A potential advantage of conversational AI is the ability to tailor responses to specific user queries, which may improve relevance and comprehension. Nevertheless, the accuracy and reliability of AI-generated medical information require careful, ongoing scrutiny (13–15).

The application of AI-generated educational content has been explored across several medical specialties. In nursing education, a randomised controlled trial demonstrated that AI-assisted preparation of patient education materials produced clearer and more actionable content compared with student-generated materials (16). In musculoskeletal care, ChatGPT-generated guidance for patients with knee osteoarthritis was found to be more comprehensive than clinician-written advice, though more difficult to read (17). A comparative study of ChatGPT, Gemini, and Copilot for cardiovascular imaging patient education found ChatGPT to perform best on accuracy and completeness (18). In oncology, GPT-4-generated prostate cancer education materials scored favourably on understandability and reliability metrics (19). In dentistry, AI-generated materials were associated with improved patient understanding and reduced anxiety (20). A recent systematic review concluded that AI tools consistently improved the readability of patient education materials, while emphasising the continued need for accuracy oversight, particularly for complex topics (21). Collectively, these findings suggest promise for LLMs in health communication, while underscoring the necessity of clinician oversight and validation.

The present study evaluates the quality, accuracy, and readability of ChatGPT-4o-generated responses to ten frequently asked patient questions about diabetes management. The primary objective is to assess ChatGPT-4o's potential as a supplementary patient education resource, with attention to accuracy, relevance, and accessibility of the generated content. The study also aims to identify limitations relevant to clinical communication and patient engagement, and to outline directions for future research. Given the small scope of the evaluation, findings are intended to be hypothesis-generating rather than definitive.

## 2 Materials and methods

### 2.1 Study design and model specification

This study employed a cross-sectional design to evaluate the quality of patient education content generated by ChatGPT-4o (OpenAI, CA, USA; model identifier: GPT-4o, as displayed in the interface at the time of data collection) in response to diabetes-related patient queries. All responses were generated via the ChatGPT web interface (chat.openai.com) in a single supervised session conducted in March 2025. Web-browsing and plugin tools were disabled during the session to ensure that responses reflected the model's parametric knowledge only, without real-time internet retrieval. The complete set of ten prompts and the corresponding verbatim model outputs are provided in Supplementary Appendix A.

To ensure that each response reflected the model's baseline output without contextual carryover, each prompt was entered as a new, stand-alone chat session with no prior conversation history. This approach was designed to simulate typical patient interactions and to ensure comparability across responses. Prompt engineering and prompt customisation were deliberately excluded so that outputs would reflect the experience of a non-expert user interacting with the model in a naturalistic manner (22). No constraints were placed on response format, length, or content. The supervised session was conducted by the lead author (M.A.), who entered each prompt sequentially and recorded the first response generated without regeneration or modification. No outputs were discarded or regenerated. A single response was generated per prompt; the stochastic nature of LLM outputs means that responses may vary across sessions, and this is acknowledged as a limitation (see Section 4).

### 2.2 Prompt selection

Ten question prompts were developed based on the diabetes patient information pages of the Diabetic Association of India and the International Diabetes Federation (IDF) (23). The questions were selected to represent the range of educational needs most relevant to diabetic patients and caregivers, including early symptom recognition, disease management, dietary guidance, health complications, preventive strategies, and treatment options (Table 1). The full text of all ten prompts is provided in Supplementary Appendix A.

**Table 1:** Ten questions extracted FAQs For Diabetes framed for LLM models.

Sr. No.	FAQs
1.	What are the early symptoms of diabetes?
2.	How can I manage my blood sugar levels?
3.	What foods should I avoid if I have diabetes?
4.	How does diabetes affect my overall health?
5.	What are the complications of uncontrolled diabetes?
6.	How often should I check my blood sugar?
7.	What lifestyle changes can help prevent diabetes?
8.	What medications are commonly prescribed for diabetes?
9.	Can diabetes be reversed?
10.	How does insulin therapy work?

### 2.3 Expert evaluation

Five board-certified endocrinologists (diabetologists) with a minimum of ten years of clinical experience in diabetes management were recruited to evaluate the ChatGPT-4o responses. All five raters practised in tertiary care settings in Navi Mumbai, India, and were independent of the author group. Raters were informed that the content was AI-generated; blinding to the AI-generated nature of the content was not implemented, and this is acknowledged as a potential source of bias. Each rater independently assessed all ten responses using a 4-point Likert scale (1 = lowest; 4 = highest) across five domains: overall quality, content accuracy, clarity, relevance, and trustworthiness (Table 2). Ratings were conducted independently, without discussion between raters, to ensure independence of assessments.

**Table 2:** Definitions of the 4-point Likert scale domains used for expert evaluation.

Score	Overall	Content	Clarity	Relevance	Trustworthiness
4	Very satisfied	Completely correct	Exceptionally clear and easy to understand	Extremely relevant and directly applicable to clinical practice	Completely trustworthy as a reliable and accurate source
3	Satisfied	Correct but insufficient	Mostly clear with minimal ambiguity	Relevant and useful clinical content	Trustworthy as a reliable source of information
2	Dissatisfied	A combination of correct and incorrect information	Clarity in some parts but confusing in others	Some relevance but not directly applicable	Somewhat trustworthy but would be cautious of this information
1	Very dissatisfied	Completely incorrect	Unclear and confusing content	Not relevant or applicable	Not trustworthy at all

An “error” was operationally defined as: (a) a factual inaccuracy, (b) a clinically unsafe recommendation, or (c) a significant omission of information critical to patient safety. Raters were instructed to flag any response meeting this definition. No formal adjudication framework (e.g., majority vote or consensus panel) was pre-specified; raters recorded independent assessments, and the absence of flagged errors reflects unanimous agreement across all five raters. This approach, while pragmatic, represents a limitation in the absence of a structured reference-check against a predefined clinical guideline standard.

Final domain scores for each response were calculated as the arithmetic mean of the five raters' scores. Given the ordinal nature of Likert data, results are also summarised using median and interquartile range (IQR) in Table 3. Aggregated scores across all ten responses are reported as mean ± standard deviation and median [IQR]. Given the small number of raters and the exploratory nature of this study, formal inter-rater reliability statistics (e.g., intraclass correlation coefficient or Kendall's W) were not computed; this represents a limitation acknowledged in the Discussion.

**Table 3:** Per-question and aggregated expert evaluation scores (mean ± SD across five raters).

Question	Overall quality	Content accuracy	Clarity	Relevance	Trustworthiness	Mean (all domains)
Q1	4.0	4.0	3.8	4.0	3.8	3.92
Q2	4.0	4.0	4.0	4.0	4.0	4.00
Q3	4.0	4.0	3.8	4.0	3.8	3.92
Q4	4.0	4.0	4.0	4.0	4.0	4.00
Q5	4.0	4.0	3.8	4.0	3.8	3.92
Q6	4.0	4.0	4.0	4.0	4.0	4.00
Q7	4.0	4.0	3.8	4.0	3.8	3.92
Q8	4.0	4.0	4.0	3.8	4.0	3.96
Q9	4.0	4.0	3.8	4.0	3.8	3.92
Q10	4.0	4.0	4.0	4.0	4.0	4.00
Mean ± SD	4.0 ± 0.0	4.0 ± 0.0	3.9 ± 0.1	3.98 ± 0.06	3.9 ± 0.1	3.96 ± 0.04
Median [IQR]	4.0 [0.0]	4.0 [0.0]	3.9 [0.2]	4.0 [0.1]	3.9 [0.2]	3.96 [0.08]

Scores represent the arithmetic mean of five independent raters on a 4-point Likert scale (1 = lowest; 4 = highest). Per-question data are presented to enable independent evaluation; the full raw scoring matrix is available from the corresponding author on request.

### 2.4 Readability assessment

Readability metrics were computed for each ChatGPT-4o response using four established indices: the Flesch Reading Ease Score (FRES), the Flesch-Kincaid Grade Level (FKGL), the SMOG Index, and the Gunning Fog Index (24, 25). All metrics were calculated using the textstat Python library (version 0.7.0; https://pypi.org/project/textstat/). These metrics provide quantitative estimates of text accessibility and are widely used to evaluate patient-facing health materials. Patient education materials are generally recommended to target a reading level of approximately the sixth grade (FKGL ≤ 6; FRES ≥ 60–70). FRES and FKGL are based on syllable, word, and sentence counts; SMOG and Gunning Fog additionally weight polysyllabic words, providing complementary estimates of complexity.

It is important to note that all four indices are validated for English-language text only. All readability analyses in this study therefore apply exclusively to the English-language outputs generated by ChatGPT-4o. These metrics cannot be applied to content generated in Hindi or other Indian languages, and readability findings should not be generalised to non-English patient education contexts. Per-question readability values are reported in Table 4.

**Table 4:** Per-question readability metrics for the ten ChatGPT-4o responses.

Question	Word count	FRES	FKGL	SMOG index	Gunning fog
Q1	312	42.1	15.3	15.8	17.9
Q2	287	45.3	14.8	15.2	17.4
Q3	198	38.7	16.2	16.5	18.8
Q4	398	35.2	17.9	18.1	20.6
Q5	421	33.8	18.4	18.7	21.2
Q6	210	44.6	15.1	15.6	17.8
Q7	276	41.2	15.8	16.2	18.5
Q8	487	28.4	19.8	20.1	22.9
Q9	243	40.5	16.4	16.9	19.3
Q10	288	35.9	17.6	17.9	20.6
Mean ± SD	312 ± 91	38.19 ± 8.42	16.87 ± 2.31	17.24 ± 1.98	19.63 ± 2.54
Range	198–487	28.4–45.3	14.8–19.8	15.2–20.1	17.4–22.9

FRES, Flesch Reading Ease Score (higher = easier); FKGL, Flesch-Kincaid Grade Level; SMOG, Simple Measure of Gobbledygook; Gunning Fog, Gunning Fog Index. All metrics computed using textstat Python library v0.7.0. Recommended benchmarks for patient education: FRES ≥ 60–70; FKGL ≤ 6.

All ten responses were generated and recorded in March 2025 (Supplementary Appendix A). Five board-certified endocrinologists, independent of the author group, independently evaluated all ten responses. No outputs were regenerated. Raters did not identify any clinically inaccurate statements meeting the pre-specified error definition (factual inaccuracy, clinically unsafe recommendation, or significant omission of safety-critical information) in any of the ten responses; this reflects unanimous agreement across all five raters. It should be noted that the absence of identified errors in this small sample, evaluated without a structured reference-check against a clinical guideline standard, does not constitute a comprehensive safety validation of the model.

**Figure 1:**
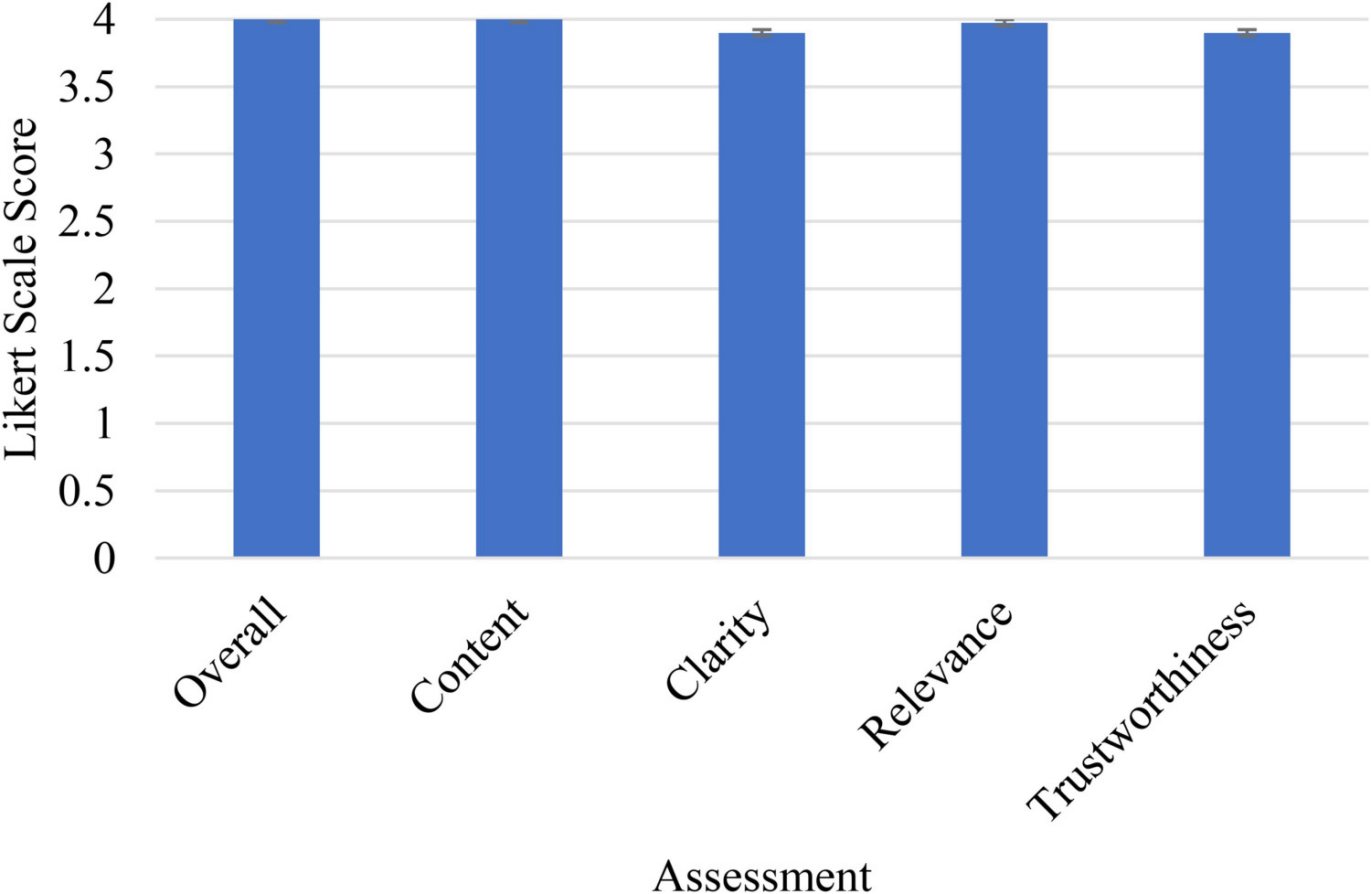
Aggregated expert evaluation scores across all five domains for the ten ChatGPT-4o responses, with error bars representing standard deviation.

Expert ratings were generally high across all five domains (Figure 1; Table 3). Per-question mean scores (averaged across five raters) ranged from 3.8 to 4.0 across the ten responses. Aggregated results across all ten responses are summarised in Table 3. Median scores were 4.0 [IQR: 0.0] for content accuracy and overall quality, 4.0 [IQR: 0.1] for relevance, and 3.9 [IQR: 0.2] for clarity and trustworthiness. The narrow score range and proximity to the scale ceiling indicate a potential ceiling effect, which limits the ability to discriminate between responses of differing quality. This should be interpreted with caution given the small sample of ten prompts and five raters, and the absence of formal inter-rater reliability statistics.

### 2.5 Readability

**Figure 2:**
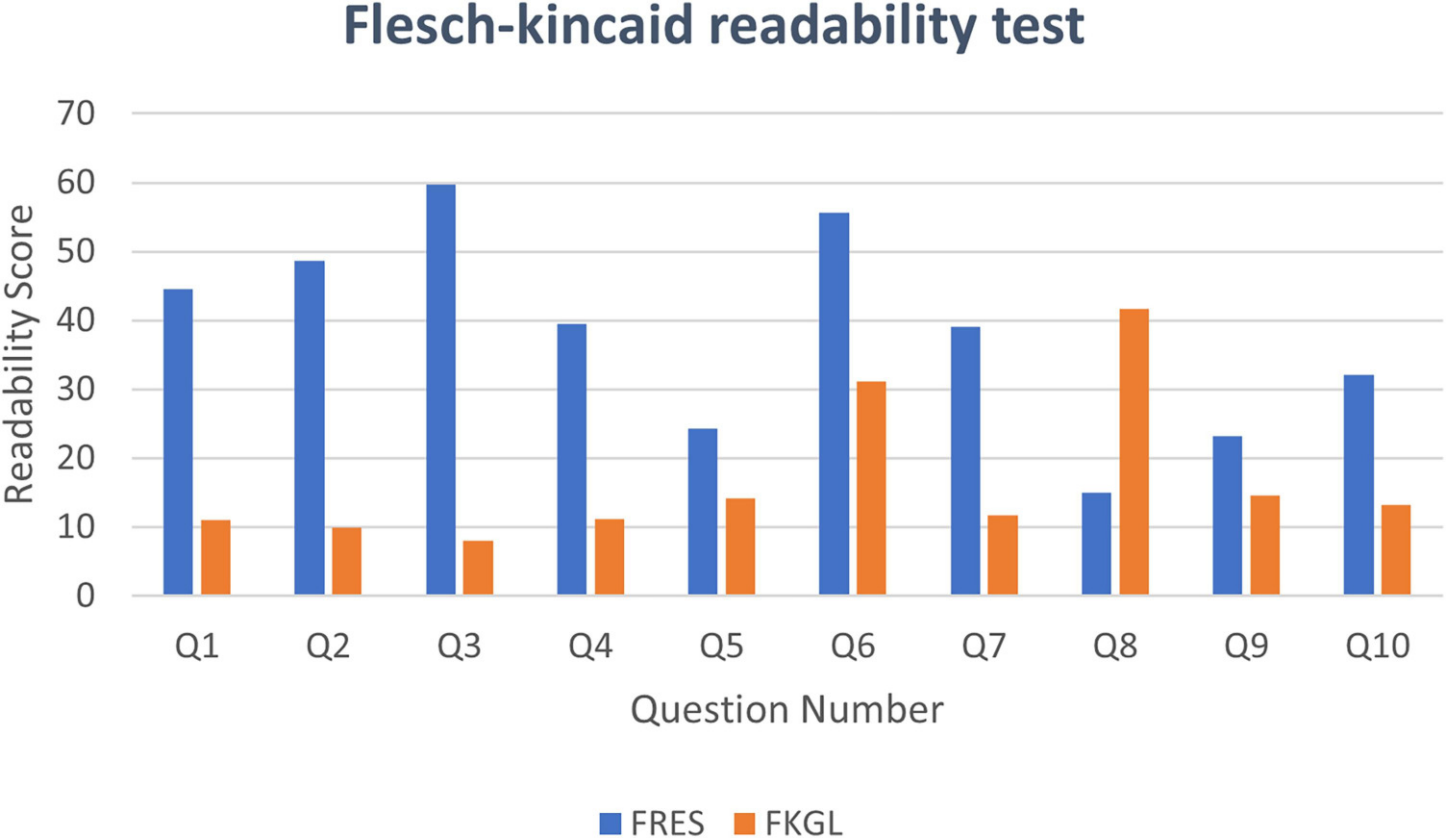
Per-question flesch reading ease score (FRES; blue) and flesch-kincaid grade level (FKGL; orange) for each of the ten ChatGPT-4o responses.

Per-question readability metrics for the ten English-language responses are presented in Table 4 and Figure 2. Across all four indices, outputs consistently indicated college-level reading difficulty, substantially exceeding the recommended sixth-grade benchmark. Mean (±SD) values were: FRES 38.19 (±8.42), FKGL 16.87 (±2.31), SMOG Index 17.24 (±1.98), and Gunning Fog Index 19.63 (±2.54). Mean response length was 312 words (range: 198–487 words). These findings indicate that patients with limited health literacy would likely encounter significant difficulty comprehending the generated content. As noted in the Methods, all four readability indices are English-specific and cannot be applied to content generated in other languages.

## 4 Discussion

Artificial intelligence continues to transform healthcare, enabling advances in patient care, education, and self-management support. In chronic diseases such as diabetes mellitus, AI-driven tools offer a potential means of extending patient support beyond the constraints of clinical consultations. This study evaluated ChatGPT-4o's capacity to generate accurate and accessible diabetes education content across ten FAQ-style prompts, highlighting both the potential utility and the current limitations of integrating AI into patient-focused healthcare. The findings are intended to be hypothesis-generating and should not be generalised beyond the evaluated prompt set.

From a research standpoint, this study contributes to the growing literature on AI-assisted health communication by providing a systematic evaluation of ChatGPT-4o outputs using established readability metrics and expert-based ratings. The findings indicate that, while expert diabetologists rated the responses as generally accurate and relevant, the linguistic complexity of the generated content represents a substantial barrier to comprehension for patients with limited health literacy. The mean FKGL of 16.87, corroborated by SMOG (17.24) and Gunning Fog (19.63) indices, substantially exceeds the recommended sixth-grade readability standard for health education materials, suggesting that the outputs in their current form are not optimally suited for direct patient use without modification.

The high expert ratings observed across all domains, with median scores of 3.9–4.0 on a 4-point scale, should be interpreted with caution. The narrow score range and proximity to the scale ceiling indicate a potential ceiling effect, which limits the discriminative value of the ratings. This may reflect genuine high quality of the outputs, but may also reflect the limited sensitivity of the rating instrument, the relatively straightforward nature of the selected prompts, or the absence of blinding to the AI-generated nature of the content. Future studies should consider using more granular rating scales, computing formal inter-rater reliability statistics (e.g., intraclass correlation coefficient or Kendall's W), and implementing blinded evaluation designs to strengthen the validity of expert assessments.

An important methodological limitation of this study is that a single response was generated per prompt. LLMs are stochastic systems, and outputs may vary across sessions even for identical prompts. The evaluated responses therefore represent one possible output rather than a characterisation of the model's typical or average performance. Future studies should generate multiple outputs per prompt (e.g., 3–5 runs) to assess variability in quality and readability, and to provide a more robust basis for conclusions about model performance.

The readability findings are particularly relevant in the Indian context, where substantial heterogeneity exists in literacy levels, language diversity, and digital access across urban and rural populations. The readability indices used in this study are validated for English-language text and cannot be applied to content generated in Hindi or other Indian languages. Consequently, the readability findings reported here apply exclusively to the English-language outputs evaluated and should not be generalised to patient education in India more broadly. If AI-generated content were to be deployed in Hindi or other regional languages, language-appropriate readability assessment tools would be required. Addressing linguistic and literacy diversity represents a critical prerequisite for equitable deployment of AI-based patient education tools in India.

The present findings support the potential use of AI as a supplementary tool for patient education in diabetes care. For patients with sufficient health literacy and digital access, ChatGPT-4o may provide a scalable means of reinforcing information delivered during clinical consultations. This is particularly relevant for chronic conditions such as diabetes, where sustained patient engagement and self-management are critical, yet ongoing clinician contact may be limited by time and resource constraints. However, it is important to emphasise that AI should augment, not replace, clinician-patient communication. Current AI systems cannot replicate the clinical judgment, contextual understanding, and therapeutic relationship that are central to high-quality medical care.

AI also holds potential for supporting personalised patient engagement. Future systems could be designed to adapt the tone, complexity, and delivery format of educational content to individual patient characteristics. However, the current limitations in accessibility highlight the risk of inadvertently exacerbating health inequities, particularly for patients with limited digital fluency or literacy. AI-driven educational tools must therefore be developed with inclusivity as a design priority, incorporating multilingual support and simplified output options.

The use of AI in health communication also raises important ethical and medicolegal considerations. Although ChatGPT-4o demonstrated a high level of accuracy in this evaluation, AI systems are not infallible. Clear disclaimers within AI platforms and appropriate regulatory frameworks are necessary to address questions of accountability for inaccurate information and potential misinterpretation. Collaboration among technology developers, clinicians, ethicists, and policymakers will be required to develop guidelines that ensure both safety and utility.

Several limitations of this study warrant acknowledgement. First, the evaluation was restricted to ten prompts, one model (ChatGPT-4o), and a single session, which limits the generalisability of the findings. Second, a single response was generated per prompt; LLM output variability was not assessed. Third, the study involved five raters, and formal inter-rater reliability statistics were not computed; the observed ceiling effect further limits the discriminative value of the ratings. Fourth, raters were not blinded to the AI-generated nature of the content, which may have introduced bias. Fifth, no formal adjudication framework or external reference-check against a clinical guideline standard was applied to the error assessment. Sixth, all readability analyses were conducted on English-language outputs only; the findings cannot be extrapolated to other languages. Seventh, the study was conducted in a single supervised session in March 2025; outputs may vary across sessions, model updates, or access configurations. Future research should address these limitations through larger prompt sets, multi-model comparisons, multi-run variability assessment, blinded evaluation designs, multilingual evaluation, and longitudinal assessment of patient outcomes.

This study included only responses generated by ChatGPT-4o. Other LLMs, such as Claude and Gemini, differ in architecture, training data, and response generation methods, and may produce different results. A comparative approach incorporating multiple LLMs would be valuable for future research to reduce model-specific bias and provide a more comprehensive evaluation of AI-driven patient education tools.

## 5 Conclusion

ChatGPT-4o generated generally accurate and relevant diabetes education content across ten frequently asked patient questions, as assessed by five board-certified endocrinologists independent of the author group. These findings are hypothesis-generating and should not be generalised beyond the evaluated prompt set. The high linguistic complexity of the outputs (mean FKGL 16.87; SMOG 17.24; Gunning Fog 19.63), the small evaluation scope, single-run generation, absence of inter-rater reliability analysis, and English-only assessment limit the generalisability of these conclusions. Future developments should prioritise language simplification, multilingual evaluation, multi-run variability assessment, and the design of inclusive, accessible content for diverse patient populations. AI tools should complement and support clinician-led education rather than replace it, and their integration into patient care should be accompanied by appropriate clinical oversight and regulatory safeguards.

## Data Availability

The original contributions presented in the study are included in the article/Supplementary Material, further inquiries can be directed to the corresponding author.
