# Supplementary material for: ChatGPT for diabetes education: potential, accuracy, and accessibility in patient support
Source: Front Digit Health. 2026 Apr 13;8:1768843. doi: 10.3389/fdgth.2026.1768843 (PMC13111439; doi:10.3389/fdgth.2026.1768843)
Supplement: Supplementary file 1 [file Datasheet1.docx]

**Appendix A**

ChatGPT 4o Responses for each question.

**1) What are the early symptoms of diabetes?**

**Response 1:** The early symptoms of diabetes can vary depending on the type (Type 1 or Type 2), but some common early warning signs include:

**Common Early Symptoms of Diabetes**

1. **Frequent Urination (Polyuria)** – Increased need to urinate, especially at night.
2. **Excessive Thirst (Polydipsia)** – Constant thirst even after drinking water.
3. **Extreme Hunger (Polyphagia)** – Increased appetite despite eating normally.
4. **Unexplained Weight Loss** – Losing weight without trying, more common in Type 1 diabetes.
5. **Fatigue and Weakness** – Feeling tired and sluggish due to the body's inability to use glucose properly.
6. **Blurred Vision** – High blood sugar levels can affect the lens of the eye, leading to temporary vision issues.
7. **Slow Healing of Wounds** – Cuts, sores, and bruises take longer to heal.
8. **Frequent Infections** – Increased susceptibility to infections, especially skin, urinary tract, or yeast infections.
9. **Tingling or Numbness in Hands and Feet** – Nerve damage (neuropathy) can begin early.
10. **Darkened Skin Patches (Acanthosis Nigricans)** – Dark, velvety patches on the neck, armpits, or groin, often seen in insulin resistance.

**When to See a Doctor**

If you experience any of these symptoms, especially in combination, it’s important to get tested for diabetes. Early diagnosis and management can prevent complications.

**2) How can I manage my blood sugar levels?**

**Response 2:** Managing blood sugar levels involves a combination of healthy eating, physical activity, proper medication (if prescribed), and lifestyle adjustments. Here are some effective strategies:

**1. Eat a Balanced Diet**

- **Choose complex carbohydrates:** Opt for whole grains (brown rice, quinoa, whole wheat), legumes, and vegetables instead of refined carbs (white bread, sugary snacks).
- **Increase fiber intake:** Foods rich in fiber, such as beans, lentils, fruits, and vegetables, slow down sugar absorption.
- **Monitor portion sizes:** Overeating, even healthy foods, can spike blood sugar.
- **Limit sugar and processed foods:** Avoid sugary drinks, pastries, candies, and processed snacks.
- **Include healthy fats and proteins:** Lean meats, fish, nuts, seeds, and olive oil help stabilize blood sugar.

**2. Stay Hydrated**

- Drink plenty of water to help flush out excess sugar from the bloodstream.
- Avoid sugary beverages like sodas and fruit juices.

**3. Exercise Regularly**

- **Aerobic exercises** like walking, jogging, cycling, or swimming help lower blood sugar.
- **Strength training** improves insulin sensitivity and glucose metabolism.
- **Consistency is key:** Aim for at least 30 minutes of moderate exercise most days.

**4. Monitor Blood Sugar Levels**

- Check your blood sugar regularly to understand how food, activity, and medication affect it.
- Keep a log to track trends and discuss them with your doctor.

**5. Manage Stress**

- Chronic stress can increase blood sugar levels.
- Practice relaxation techniques like deep breathing, meditation, or yoga.

**6. Get Enough Sleep**

- Poor sleep can lead to insulin resistance.
- Aim for 7-9 hours of quality sleep per night.

**7. Take Medications as Prescribed**

- If you have diabetes or prediabetes, follow your doctor’s medication plan.
- Never adjust doses without consulting a healthcare professional.

**8. Avoid Smoking and Alcohol in Excess**

- Smoking and excessive alcohol can raise blood sugar levels.
- If you drink, do so in moderation and pair alcohol with food.

**3) What foods should I avoid if I have diabetes?**

**Response 3:** If you have diabetes, managing your blood sugar levels is crucial. Here are the foods you should avoid or limit:

**High-Carbohydrate Foods (Refined & Processed)**

1. **White Bread, Pasta, and Rice** – Opt for whole grains instead.
2. **Sugary Cereals** – Choose high-fiber, low-sugar options.
3. **Pastries, Cakes, and Doughnuts** – Loaded with refined sugar and unhealthy fats.

**Sugary Foods and Drinks**

1. **Sodas and Sugary Juices** – High in fructose, causing insulin resistance.
2. **Candy and Sweets** – Can lead to rapid blood sugar spikes.
3. **Flavored Yogurt** – Often contains hidden sugars; prefer plain yogurt.

**Unhealthy Fats**

1. **Fried Foods (French Fries, Fried Chicken, etc.)** – High in trans fats and calories.
2. **Processed Meats (Sausages, Hot Dogs, Bacon)** – High in sodium and unhealthy fats.
3. **Margarine and Shortening** – Contain trans fats, harmful to heart health.

**High-Glycemic Fruits & Vegetables**

1. **Potatoes (especially mashed or fried)** – High glycemic index.
2. **Ripe Bananas, Grapes, and Pineapples** – Can spike blood sugar quickly.
3. **Canned Fruits in Syrup** – Added sugars increase carb content.

**Dairy and Dairy Alternatives**

1. **Flavored or Sweetened Milk Alternatives** – Often contain added sugar.
2. **Full-Fat Dairy Products (if overconsumed)** – May contribute to insulin resistance.

**Alcoholic Beverages**

1. **Beer, Sweet Wines, and Cocktails** – High in carbs and sugars.
2. **Excessive Alcohol** – Can interfere with blood sugar regulation.

**Hidden Sugar Sources**

1. **Ketchup, BBQ Sauce, and Salad Dressings** – Often contain added sugar.
2. **Granola Bars and Energy Bars** – Seem healthy but can be sugar-laden.
3. **Fast Food and Processed Foods** – High in refined carbs, sodium, and unhealthy fats.

**Best Practices**

- Focus on **whole, unprocessed foods** like lean proteins, healthy fats, and fiber-rich carbs.
- Choose **low-GI foods** like **leafy greens, whole grains, and nuts**.
- Drink **water, unsweetened tea, or black coffee** instead of sugary beverages.
- Read **nutrition labels** to check for **hidden sugars** (corn syrup, dextrose, etc.).

**4) How does diabetes affect my overall health?**

**Response 1:** Diabetes can have widespread effects on your overall health, impacting various organs and systems in your body. Here’s how:

1. Cardiovascular System

- Increases the risk of heart disease, high blood pressure, and stroke due to high blood sugar damaging blood vessels.
- Leads to atherosclerosis (hardening of arteries), increasing the risk of heart attacks.

2. Nervous System (Neuropathy)

- Peripheral neuropathy: Causes tingling, numbness, or pain in the hands and feet.
- Autonomic neuropathy: Affects internal organs, leading to digestive problems, bladder issues, and difficulty regulating blood pressure.

3. Kidneys (Diabetic Nephropathy)

- High blood sugar damages kidney filtration units, leading to chronic kidney disease or even kidney failure.

4. Eyes (Diabetic Retinopathy)

- Increases the risk of vision loss, cataracts, and glaucoma due to damage to the retina.
- Can cause blindness if untreated.

5. Immune System (Increased Infections)

- Slows wound healing and increases susceptibility to infections, including skin infections, UTIs, and gum disease.
- Higher risk of diabetic foot ulcers, which can lead to amputation in severe cases.

6. Digestive System

- Can cause gastroparesis, leading to slow stomach emptying, nausea, bloating, and acid reflux.
- Increases the risk of fatty liver disease.

7. Mental Health

- Higher risk of depression, anxiety, and cognitive decline due to fluctuating blood sugar levels and stress.

8. Hormonal and Sexual Health

- Can lead to erectile dysfunction in men and reduced libido in both men and women.
- May cause menstrual irregularities in women.

9. Skin and Oral Health

- Prone to dry skin, bacterial and fungal infections.
- Increased risk of gum disease (periodontitis), leading to tooth loss.

10. Increased Risk of Stroke and Dementia

- Diabetes increases the risk of stroke by damaging blood vessels in the brain.
- Higher risk of Alzheimer’s disease and vascular dementia due to poor blood flow to the brain.

What Can You Do?

- Maintain blood sugar control through a healthy diet, regular exercise, and prescribed medications.
- Monitor blood pressure and cholesterol to reduce cardiovascular risks.
- Regular check-ups with your doctor, eye exams, and kidney function tests.
- Practice good foot and oral care to prevent infections and complications.

Managing diabetes effectively can significantly reduce the risk of complications and improve your overall well-being.

**5)** **What are the complications of uncontrolled diabetes?**

**Response 5:** Uncontrolled diabetes can lead to several serious **short-term and long-term complications**, affecting multiple organs and systems in the body. These complications arise due to prolonged high blood sugar levels, which damage blood vessels, nerves, and organs. Here are some key complications:

**Short-Term Complications**

1. **Diabetic Ketoacidosis (DKA)** – More common in Type 1 diabetes, occurs due to insulin deficiency leading to excess ketone production, causing severe dehydration, acid buildup, and potential coma.
2. **Hyperosmolar Hyperglycemic State (HHS)** – More common in Type 2 diabetes, causes extremely high blood sugar leading to severe dehydration, confusion, and coma.
3. **Hypoglycemia (Low Blood Sugar)** – Can occur due to excessive insulin, missed meals, or excessive exercise, leading to dizziness, confusion, seizures, or unconsciousness.

**Long-Term Complications**

**1. Cardiovascular Complications**

- **Heart Disease & Stroke** – High blood sugar damages blood vessels, increasing the risk of heart attacks, strokes, and high blood pressure.
- **Peripheral Artery Disease (PAD)** – Narrowed arteries reduce blood flow to limbs, leading to pain, poor wound healing, and increased risk of amputation.

**2. Neuropathy (Nerve Damage)**

- **Peripheral Neuropathy** – Tingling, numbness, pain, or weakness in hands and feet, increasing the risk of ulcers and infections.
- **Autonomic Neuropathy** – Affects involuntary functions, leading to issues like digestive problems (gastroparesis), bladder dysfunction, erectile dysfunction, and abnormal heart rate/blood pressure regulation.

**3. Nephropathy (Kidney Damage)**

- **Diabetic Nephropathy** – High blood sugar damages kidney filters, leading to protein loss in urine, high blood pressure, and eventually kidney failure requiring dialysis or transplant.

**4. Retinopathy & Eye Problems**

- **Diabetic Retinopathy** – Damage to the retinal blood vessels, causing vision loss or blindness.
- **Macular Edema** – Swelling in the macula, leading to blurred vision.
- **Cataracts & Glaucoma** – Increased risk of these eye conditions due to prolonged high blood sugar.

**5. Diabetic Foot Complications**

- **Poor Circulation & Neuropathy** – Leads to slow-healing wounds, ulcers, and infections, increasing the risk of amputation.
- **Charcot Foot** – Deformity due to nerve damage affecting foot structure.

**6. Skin Infections & Poor Wound Healing**

- **Bacterial & Fungal Infections** – High sugar levels promote infections like cellulitis, fungal infections (candida), and abscesses.
- **Diabetic Dermopathy** – Dark patches or shin spots due to blood vessel damage.

**7. Mental Health Issues**

- **Depression & Anxiety** – Chronic illness stress can contribute to mental health challenges.
- **Cognitive Decline** – Increased risk of Alzheimer’s disease and dementia due to prolonged high blood sugar levels.

**Prevention & Management**

- Maintain **tight blood sugar control** through medication, diet, and lifestyle changes.
- Regular **monitoring of blood sugar levels**.
- Routine **check-ups** with healthcare providers for early detection of complications.
- **Exercise** and **healthy eating** to manage weight and improve insulin sensitivity.
- Proper **foot care** to prevent ulcers and infections.

**6)** **How often should I check my blood sugar?**

**Response 6:** How often you should check your blood sugar depends on several factors, such as whether you have diabetes, your treatment plan, and your doctor's recommendations. Here’s a general guide:

If You Have Type 1 Diabetes:

- Before meals and snacks
- Before and after exercise
- Before bed
- Occasionally at night (e.g., 2-3 AM)
- More frequently if you’re sick, stressed, or adjusting medications

If You Have Type 2 Diabetes:

- Once or twice a day (if on oral medications or lifestyle management)
- Before meals and at bedtime (if on insulin)
- More frequently if starting a new medication or changing your routine

If You Don't Have Diabetes:

- Occasionally, if at risk (e.g., family history, obesity, or symptoms like fatigue and excessive thirst)
- During annual check-ups if you have prediabetes or metabolic concerns

**7) What lifestyle changes can help prevent diabetes?**

**Response 7:** Preventing diabetes, particularly type 2 diabetes, involves making long-term lifestyle changes that promote overall health. Here are some key lifestyle modifications that can help prevent or delay diabetes:

1. Maintain a Healthy Diet

- Choose Whole Foods: Opt for whole grains, lean proteins, and plenty of vegetables.
- Reduce Sugar and Refined Carbs: Avoid sugary drinks, white bread, and processed foods.
- Increase Fiber Intake: High-fiber foods like lentils, beans, whole grains, and vegetables improve blood sugar control.
- Healthy Fats: Include sources of unsaturated fats such as nuts, seeds, avocados, and olive oil.
- Portion Control: Eating in moderation prevents insulin resistance and weight gain.

2. Stay Physically Active

- Exercise Regularly: Aim for at least 150 minutes of moderate exercise per week (e.g., brisk walking, cycling, swimming).
- Strength Training: Helps improve insulin sensitivity and maintain muscle mass.
- Reduce Sitting Time: Avoid prolonged inactivity; take breaks to walk or stretch.

3. Maintain a Healthy Weight

- Lose Excess Weight: Even a 5-10% weight loss can significantly lower the risk of diabetes.
- Avoid Crash Diets: Focus on sustainable lifestyle changes rather than extreme diets.

4. Monitor and Manage Stress

- Practice Relaxation Techniques: Meditation, deep breathing, and yoga help reduce stress-related hormone imbalances.
- Get Enough Sleep: Aim for 7-9 hours of quality sleep per night to prevent insulin resistance.

5. Stay Hydrated

- Drink More Water: Replaces sugary drinks and helps regulate blood sugar levels.
- Limit Sugary Beverages: Avoid sodas, fruit juices, and sweetened teas.

6. Avoid Harmful Habits

- Quit Smoking: Increases insulin resistance and diabetes risk.
- Limit Alcohol: Excessive alcohol intake can contribute to weight gain and blood sugar fluctuations.

7. Regular Health Checkups

- Monitor Blood Sugar Levels: Especially if you have a family history of diabetes.
- Check Blood Pressure & Cholesterol: Keeping them under control reduces diabetes risk.

8. Get Enough Vitamin D

- Sun Exposure & Supplements: Helps regulate insulin function.

By adopting these lifestyle changes, you can **significantly reduce your risk of diabetes** and improve overall health.

**8)** **What medications are commonly prescribed for diabetes?**

**Response 8:** Medications commonly prescribed for **diabetes** depend on the **type of diabetes** (Type 1, Type 2, or gestational diabetes) and individual patient needs. Here’s a breakdown of the most commonly prescribed **anti-diabetic medications**:

**1. Type 1 Diabetes**

Type 1 diabetes requires **insulin therapy**, as the pancreas does not produce insulin.

**Insulin Types:**

- **Rapid-acting Insulin** (works within 15 minutes, lasts 2-4 hours)
  - Insulin **Lispro** (Humalog)
  - Insulin **Aspart** (NovoLog)
  - Insulin **Glulisine** (Apidra)
- **Short-acting Insulin** (starts in 30 minutes, lasts 3-6 hours)
  - Regular insulin (Humulin R, Novolin R)
- **Intermediate-acting Insulin** (starts in 1-2 hours, lasts 12-18 hours)
  - **NPH insulin** (Humulin N, Novolin N)
- **Long-acting Insulin** (lasts 24 hours or more)
  - Insulin **Glargine** (Lantus, Basaglar, Toujeo)
  - Insulin **Detemir** (Levemir)
  - Insulin **Degludec** (Tresiba)
- **Premixed Insulin** (combination of rapid/short-acting and intermediate-acting insulin)
  - Humalog Mix 75/25, Novolog Mix 70/30

**2. Type 2 Diabetes**

Type 2 diabetes is often managed with **oral medications** and sometimes **injectable non-insulin drugs** or **insulin**.

**Oral Medications:**

1. **Biguanides** (First-line treatment)
   - **Metformin** (Glucophage, Fortamet, Riomet) – reduces liver glucose production and improves insulin sensitivity.
2. **Sulfonylureas** (Stimulate insulin release)
   - **Glimepiride** (Amaryl)
   - **Glipizide** (Glucotrol)
   - **Glyburide** (Diabeta, Micronase)
3. **Meglitinides** (Short-acting insulin secretagogues)
   - **Repaglinide** (Prandin)
   - **Nateglinide** (Starlix)
4. **Thiazolidinediones (TZDs)** (Improve insulin sensitivity)
   - **Pioglitazone** (Actos)
   - **Rosiglitazone** (Avandia)
5. **DPP-4 Inhibitors** (Increase incretin levels, reducing blood sugar)
   - **Sitagliptin** (Januvia)
   - **Saxagliptin** (Onglyza)
   - **Linagliptin** (Tradjenta)
   - **Alogliptin** (Nesina)
6. **SGLT2 Inhibitors** (Prevent glucose reabsorption in kidneys)
   - **Empagliflozin** (Jardiance)
   - **Canagliflozin** (Invokana)
   - **Dapagliflozin** (Farxiga)
   - **Ertugliflozin** (Steglatro)
7. **Alpha-glucosidase Inhibitors** (Delay carbohydrate absorption)
   - **Acarbose** (Precose)
   - **Miglitol** (Glyset)
8. **Bile Acid Sequestrants** (Lower glucose levels indirectly)
   - **Colesevelam** (Welchol)

**Injectable Non-Insulin Medications:**

1. **GLP-1 Receptor Agonists** (Increase insulin secretion, slow digestion)
   - **Liraglutide** (Victoza, Saxenda)
   - **Dulaglutide** (Trulicity)
   - **Semaglutide** (Ozempic, Rybelsus (oral))
   - **Exenatide** (Byetta, Bydureon)
   - **Lixisenatide** (Adlyxin)
2. **Amylin Analogues** (Used alongside insulin to control post-meal blood sugar)
   - **Pramlintide** (Symlin)

**3. Gestational Diabetes (During Pregnancy)**

- **Insulin** (preferred treatment)
- **Metformin** (used in some cases)
- **Glyburide** (sometimes used but less preferred)

**Combination Medications**

Some patients take combination drugs to simplify their regimen:

- **Metformin + DPP-4 inhibitor** (Janumet = Sitagliptin + Metformin)
- **Metformin + SGLT2 inhibitor** (Invokamet = Canagliflozin + Metformin)
- **Metformin + Sulfonylurea** (Glucovance = Glyburide + Metformin)
- **GLP-1 Agonist + Basal Insulin** (Xultophy = Liraglutide + Insulin Degludec)

**9) Can diabetes be reversed?**

**Response 9:** Diabetes reversal depends on the type of diabetes and various individual factors:

**Type 1 Diabetes:**

- **Not reversible**: Type 1 diabetes is an autoimmune condition where the body’s immune system attacks insulin-producing beta cells in the pancreas. Currently, there is no cure, but management through **insulin therapy, diet, and lifestyle** can help maintain normal blood sugar levels.

**Type 2 Diabetes:**

- **Potentially reversible**: Type 2 diabetes is largely influenced by lifestyle factors, and in some cases, people can achieve remission through:
  - **Weight loss**: Studies show that **losing 10-15% of body weight** can lead to diabetes remission in some cases.
  - **Dietary changes**: Low-carb, Mediterranean, and plant-based diets can improve insulin sensitivity.
  - **Intermittent fasting**: Some evidence suggests that fasting periods can help reset insulin response.
  - **Exercise**: Regular physical activity improves glucose metabolism.
  - **Bariatric surgery**: Some individuals who undergo weight-loss surgery experience long-term remission.

**Gestational Diabetes:**

- **Usually reversible**: This type of diabetes occurs during pregnancy and typically resolves after childbirth. However, it increases the risk of developing Type 2 diabetes later in life.

**Key Takeaway:**

- **Remission is possible** in Type 2 diabetes through weight loss and lifestyle changes, but ongoing management is crucial.
- **Regular monitoring is essential**, as blood sugar levels can rise again if lifestyle changes are not maintained.

**10) How does insulin therapy work?**

**Response 10:** Insulin therapy is a treatment used to manage diabetes mellitus, particularly for people with type 1 diabetes and some individuals with type 2 diabetes when oral medications or lifestyle changes are not sufficient. Insulin is a hormone produced by the pancreas that helps regulate blood sugar (glucose) levels.

1. Role of Insulin in the Body

- Insulin allows glucose to enter cells, where it is used for energy.
- It lowers blood sugar levels by signaling the liver to store excess glucose as glycogen.
- In diabetes, either the body does not produce insulin (Type 1 Diabetes) or the body’s cells do not respond properly to insulin (Type 2 Diabetes).
- Without insulin, blood glucose levels become too high (hyperglycemia), leading to complications like nerve damage, kidney failure, and cardiovascular disease.

2. Types of Insulin Therapy

Insulin therapy involves different types of insulin based on how fast they act and how long they last:

| Type of Insulin | Onset (Starts Working) | Peak Action | Duration |
| --- | --- | --- | --- |
| Rapid-acting (e.g., Lispro, Aspart) | 10-15 min | 30-90 min | 3-5 hours |
| Short-acting (Regular Insulin) | 30 min-1 hour | 2-4 hours | 6-8 hours |
| Intermediate-acting (NPH) | 1-2 hours | 4-12 hours | 12-18 hours |
| Long-acting (e.g., Glargine, Detemir) | 1-2 hours | No peak | Up to 24 hours |
| Ultra-long-acting (e.g., Degludec) | 1-2 hours | No peak | Up to 42 hours |

3. Methods of Insulin Delivery

Insulin can be administered in different ways:

1. Insulin Injections (via syringe or insulin pen) – Most common method.
2. Insulin Pumps – Delivers a continuous dose of insulin throughout the day.
3. Inhaled Insulin – A newer method (e.g., Afrezza) for rapid-acting insulin.
4. Artificial Pancreas (Closed-Loop System) – A more advanced system that adjusts insulin automatically.

4. Insulin Therapy Regimens

- Basal-Bolus Therapy (Multiple Daily Injections - MDI):
  - Basal insulin (long-acting) taken once or twice daily.
  - Bolus insulin (rapid-acting) taken before meals to cover carbohydrate intake.
- Twice-Daily Insulin Therapy: Mix of short- and intermediate-acting insulin given morning and night.
- Continuous Subcutaneous Insulin Infusion (CSII): Used with insulin pumps.

5. Monitoring & Adjusting Insulin Doses

- Blood Glucose Monitoring: Patients use glucometers or continuous glucose monitors (CGMs) to track sugar levels.
- Carbohydrate Counting: Helps determine insulin dose before meals.
- Exercise & Lifestyle Adjustments: Physical activity affects insulin sensitivity, requiring dosage adjustments.

6. Risks & Side Effects

- Hypoglycemia (Low Blood Sugar) – Can occur if too much insulin is taken or food intake is insufficient.
- Weight Gain – Due to improved glucose absorption and energy storage.
- Insulin Resistance – In type 2 diabetes, the body may need higher doses over time.
- Lipodystrophy – Fat tissue changes at injection sites if the same spot is used repeatedly.

7. Who Needs Insulin Therapy?

- Type 1 Diabetes: Must use insulin lifelong.
- Type 2 Diabetes: If oral medications fail, or during illness, pregnancy, or surgery.
- Gestational Diabetes: Sometimes needed if blood sugar is not controlled with diet alone.

Conclusion

Insulin therapy is a lifesaving treatment for diabetes, helping maintain normal blood sugar levels and preventing complications. Proper monitoring, individualized dosing, and lifestyle adjustments ensure safe and effective use.
